# Can drug repurposing stop “chase and run” between aldehydes and reactive sulfur species in anti-cancer therapy?

**DOI:** 10.18632/oncotarget.26170

**Published:** 2018-10-02

**Authors:** Makoto Suematsu

**Affiliations:** Makoto Suematsu: Department of Biochemistry, Keio University School of Medicine, Shinjuku-ku, Tokyo, Japan

**Keywords:** trans-sulfuration pathway, polysulfides, hydrogen sulfide, Warburg effect, cancer metabolism

Cancer cells accelerate glycolysis to maintain ATP and other metabolites which are necessary to maintain their survival and chemoresistance. Glutathione (GSH) is the major antioxidant in living organisms and has multiple functions including maintenance of intracellular redox homeostasis. Cancer cells produce reactive oxygen species (ROS) that results from mitochondrial dysfunction, aberrant metabolism, and frequent genetic mutations. Such circumstances cause accumulation of large amounts of oxidized molecules including proteins, DNA and lipids. Therefore, cancer cells require active synthesis and recycling of GSH under regulating the balance of glucose utilization between glycolysis and pentose phosphate pathway which is necessary to synthesize NADPH and nucleic acid precursors [1]. Excess GSH results in tumor progression as well as therapeutic resistance.

Ferroptosis has recently been identified as a novel type of regulated cell death caused by severe oxidative stress and subsequent lipid peroxidation against which GSH-dependent antioxidant system protects. Inhibition of xCT cystine transporter or the GSH-dependent enzyme glutathione peroxidase 4 has been shown to induce ferroptosis in cancer cells [2]. Since mechanisms for ferroptosis are apoptosis-independent [2], the ferroptosis-inducing cancer therapy is expected to be effective even for the refractory cancers with resistance against apoptosis. Recently, clinical studies using the xCT inhibitors have been conducted in human malignancies [3, 4]. On the other hand, cancer cells have been reported to often acquire the resistance to GSH-depletion through the activation of pro-survival pathway [5]. Thus, the activation of such alternative pathways leads to cancer survival, and thus may serve as obstacles for the development of xCT-targeted therapy in future.

In this issue of *Oncotarget*, Okazaki et al. performed a synthetic lethal screen of a drug library to identify agents that sensitize the GSH deficiency-resistant cancer cells to the xCT inhibitor sulfasalazine and identified the oral anesthetic dyclonine. Dyclonine possesses a unique structure responsible for covalent inhibition of aldehyde dehydrogenase enzymes (ALDHs) [6]. Treatment with dyclonine induced intracellular accumulation of the toxic aldehyde 4-hydroxynonenal (4-HNE) in a cooperative manner with sulfasalazine in the xCT inhibitor resistant cancer cells [6]. They also showed that elevation of ALDH3A1 expression contributes to the resistance to sulfasalazine in cancer cells and the combination of dyclonine and sulfasalazine cooperatively suppressed the growth of highly ALDH3A1-expressing tumors that were resistant to sulfasalazine monotherapy. Besides aldehyde accumulation, to be noted is that ALDH3A1 inhibition might cancel bioactivation of nitrite to generate nitric oxide which also plays a protective role for cancer survival [7]. Furthermore, chronic accumulation of aldehydes as electrophiles causes Nrf2 activation and thus activate multiple enzyme systems including cystathionine β-synthase and/or cystathionine γ-lyase in parallel with serine/glycine cleavage systems that provide carbon units from 3-phosphoglycerate in glycolysis towards trans-sulfuration pathway to enhance anti-oxidative hypotaurine or nucleophilic polysulfides that cancel aldehydes and varied electrophiles to contribute to cancer survival and persistent drug resistance [8].

In this context, of importance is that an engineered human enzyme cyst(e)inase which can degrade cystine/cysteine systemically has been developed [9]. The clinical use of xCT inhibitors or cyst(e)inase is expected to effectively deplete GSH, hypotaurine and polysulfides from tumor tissues. Therefore, the identification of drugs inducing synthetic lethality in cancer cells with GSH deficiency is useful for the effective cancer therapy using GSH-depleting agents.

## References

[1] Yamamoto T (2014). Nat Commun.

[2] Yang WS (2014). Cell.

[3] Shitara K (2017). Gastric Cancer.

[4] Otsubo K (2017). Cancer Sci.

[5] De Nicola M (2014). Front Pharmacol.

[6] Okazaki S (2018). Oncotarget.

[7] Lin S (2013). Nitric Oxide.

[8] Shiota M (2018). Nat Commun.

[9] Cramer SL (2017). Nat Med.

